# Mothers' Views on the Use of Oral Glucose for Pain Relief in a Neonatal Intensive Care Unit

**DOI:** 10.1111/nicc.70318

**Published:** 2026-01-08

**Authors:** Anna‐Kaija Palomaa, Eeva Talus, Sirpa Keskitalo‐Leskinen, Tarja Pölkki

**Affiliations:** ^1^ Research Unit of Health Sciences and Technology, Faculty of Medicine University of Oulu Oulu Finland; ^2^ Medical Research Center Oulu, Oulu University Hospital University of Oulu Oulu Finland; ^3^ Pediatric and Neonatal Intensive Care Unit Oulu University Hospital Oulu Finland

**Keywords:** family‐centred care, glucose, newborn, pain, parents

## Abstract

**Background:**

Involving mothers in their infant's pain management is an essential part of family‐centred care in a neonatal intensive care unit (NICU). Healthcare professionals commonly use sweet solutions, such as oral glucose, to relieve pain in infants during heel lances and other procedures. However, there is a lack of knowledge about how mothers perceive the use of oral glucose for pain management.

**Aim:**

To describe mothers' views on the use of oral glucose for neonatal pain relief.

**Study Design:**

A qualitative descriptive study was conducted in a neonatal intensive care unit in Finland between May 2023 and May 2024. The participants were mothers (*n* = 25), recruited from a randomised controlled trial (RCT) involving newborns. Data were collected through semi‐structured interviews based on an interview guide, and analysed using content analysis.

**Findings:**

Mothers' views on the use of glucose for neonatal pain relief consisted of four main categories: ‘Varying perceptions of acceptability’, ‘An easy‐to‐implement method’, ‘Contradictory opinions on effectiveness’ and ‘Another method would be better’.

**Conclusions:**

Mothers found glucose to be an easy‐to‐use method for neonatal pain relief, but their opinions on its effectiveness varied. They recommended combining glucose with another method or adopting a mother‐led approach to improve pain management.

**Relevance to Clinical Practice:**

Healthcare professionals should recognise the individual nature of each mother's perception of glucose as a method of newborn pain management and consider this perspective when providing counselling and planning pain relief methods.

## Impact Statements

- What is known about the topic
  ○ Sweet solutions are a widely used and effective pain relief method that healthcare professionals commonly use in NICUs.
  ○ Parents want to be involved in their newborn's pain management. However, they rarely use sweet solutions for pain relief in the NICU.
- What this paper adds
  ○ Mothers have different perceptions regarding the acceptability and effectiveness of using glucose to manage newborn pain in NICU.
  ○ Mothers have a desire to contribute to their infant's comfort during painful procedures by fostering a sense of safety. A part of this involvement, they propose the use of breast milk as an alternative to glucose for pain relief.

## 1 Introduction

Infants admitted to neonatal intensive care unit (NICU) undergo an average of more than seven painful procedures daily [1]. Repeated exposure to untreated procedural pain causes immediate physiological instability [2] and can lead to long‐term adverse consequences [3]. The negative effects of neonatal pain also extend to the family. Seeing the infant in pain and being unable to comfort the infant causes significant stress for parents [4], and can increase the mother's risk of post‐traumatic disorder after the infant is discharged [5].

### 1.1 Background

Parental involvement in infants' pain management is an essential component of family‐centred care [6], and has become one of the main areas of research in neonatal pain management [7, 8, 9]. Previous studies show that a wide range of non‐pharmacological pain relief methods can be delivered by parents [10], and parental pain relief provides effective pain alleviation for infants [11]. Most parents want to be actively involved in their infant's pain management [12]. This involvement may encompass the provision of pain relief, participation in decision‐making and advocacy for preferred pain relief methods [8, 12]. There is evidence that parental involvement not only improves infant pain management [13] but also supports parental well‐being. A recent study showed that parental involvement in pain management can relieve parents' stress and make them feel useful and reassured [14]. On the other hand, negative feelings have also been reported; some parents consider pain management to be the responsibility of professionals and feel distressed if they have to be involved in pain management [8, 10].

Sweet solutions such as sucrose and glucose are one of the most frequently studied pain relief methods for preterm and term infants [15] and have long been considered the gold standard for neonatal pain management [16] in several guidelines [17]. Strong evidence from systematic reviews suggests that sucrose [18] and glucose [19] are effective pain relief methods for single minor procedures such as heel lances and venipuncture without serious side effects [18, 19]. This may be why sweet solutions are the most used procedural pain relief method in hospitals [20].

Based on early studies, pain management with sweet solutions is mostly delivered by nurses. They report using sucrose or glucose frequently for pain relief in infants [21, 22], while mothers are less likely to use sweet solutions for pain relief in their newborns [23, 24]. It is possible that in some hospitals, sweet solutions are considered professional‐administered medication [25]. Researchers also appear uncertain about the role of parents in glucose‐based pain management [8, 10]. Meanwhile, there is limited knowledge on how mothers perceive the use of sweet solutions for their critically ill infant's pain relief.

## 2 Aim

The aim of this study was to describe mothers' views on the use of oral glucose for neonatal pain relief in a NICU.

## 3 Design and Methods

The study used a descriptive qualitative study design to gain a deeper understanding of how mothers perceive the use of oral glucose (sweet solution) for pain relief in critically ill newborn infants [26]. The research method followed was semi‐structured interviews, which allowed mothers to openly express their personal thoughts and views [27]. The article follows the COREQ checklist (Table S2) for reporting qualitative studies [28].

### 3.1 Setting and Sample

The study was conducted in a Level III NICU in Finland between May 2023 and May 2024. The unit has 20 beds for premature and sick newborns, including 12 intensive care beds and 8 beds for newborns requiring specialised care. The unit adheres to the principles of family‐centred care and welcomes parents as part of their infant's care team. The intensive care rooms are open and do not have beds for parents. In this unit, a 30% oral glucose solution is a standard intervention for procedural pain, either alone or in combination with other pain relief methods. The participants were recruited among mothers whose newborns had previously taken part in a randomised controlled trial (RCT) evaluating the effectiveness of non‐pharmacological methods for pain relief (ClinicalTrials.gov NCT04967118). All mothers who met the following inclusion criteria were recruited: (a) being the infant's birth mother, (b) being Finnish speaking and (c) being willing to participate in the study and giving written informed consent for participation.

### 3.2 Data Collection Tools and Methods

The semi‐structured individual interviews were conducted between May 2023 and May 2024, excluding the summer months of June to August. The first author conducted face‐to‐face interviews. The audio‐recorded interviews were conducted in the NICU with only the interviewer, the mother and, in some cases, the infant present. At the beginning of the interview, the researcher explained the themes of the interviews (Table 1), collected background information using a structured questionnaire and invited the mother to ask if anything was unclear. The themes of the interview were adapted from previous literature [12, 29] and pre‐tested on mothers (*n* = 3) who did not take part in the study.

**TABLE 1:** The interview guide.

1. Pain experienced by the newborn infant and the mother's previous involvement in pain relief
Could you tell me what your baby is like?
Could you tell me if your baby has had any pain during intensive care? When? What kind of pain?
Could you tell me if you were involved in your baby's pain relief before this study? How was your experience?
2. Oral glucose solution for neonatal pain relief
Could you tell me if you have any previous experience using glucose to relieve your baby's pain?
Could you tell me how you felt about using oral glucose alone to relieve your baby's pain?
Could you tell me how you think the baby experienced the oral glucose solution alone for pain relief?

It took 10 months to collect the mothers' interview data. The sample size was determined based on the saturation of the data, which meant that no new codes were identified [30]. Saturation was confirmed by conducting five additional interviews, resulting in a sample size of 25 mothers. The interviews lasted 14 to 30 min.

### 3.3 Data Analysis

The mothers' and infants' background characteristics were analysed using descriptive statistics. The interview data was analysed using inductive content analysis [30]. The analysis was guided by the research questions and was conducted simultaneously with data collection. Initially, the first author transcribed the interviews verbatim and read them several times to familiarise herself with the data. The unit of analysis chosen was part of a sentence, a sentence or a phrase. The next step was to organise the data. Original expressions that concerned the aim of the research were collected on a coding sheet and were simplified by removing the filler words and converting dialect words into common language. The simplified expressions (*n* = 122) were open coded by underlining different colours, writing notes in margins and using headings to describe the dimensions of the content of the data. The expressions were then grouped into subcategories based on similarities and differences. Each subcategory was named using words specific to the content. The abstraction process was continued by combining subcategories into generic categories and finally into main categories. Throughout the analysis, the main categories were examined in relation to the research questions by moving back and forth between the data, codes and subcategories. The first author conducted the initial categorisation, but the whole research team participated in evaluating the categorisation and creating the final categories.

### 3.4 Ethical and Institutional Approvals

The study was conducted in accordance with the Declaration of Helsinki and the guidelines of the Finnish National Board on Research Integrity [31, 32]. The Regional Medical Research Ethics Committee of the North Ostrobothnia Wellbeing Services County approved this study as one part of a randomised controlled clinical trial (RCT) (ref: 296/2018) in March 2023. Mothers were recruited into the study simultaneously with their children into the RCT. The first author involved in the data collection approached mothers face‐to‐face. Mothers were given verbal and written information about the study including the contact information of the researcher responsible for this. They signed a document giving informed consent prior to taking part in the study. The mothers were aware that they had the right to withdraw from the study at any time.

### 3.5 Rigour and Reflexivity

The trustworthiness of this study was evaluated based on credibility, dependability, conformability and transferability [33]. The study's credibility was enhanced by its careful design, detailed analysis description, systematically reported results and diverse research team (Table S1). To improve dependability, the first author conducted all interviews according to the interview guide. The study's conformability was strengthened by using reflexivity to promote transparency and consistency throughout the analysis process. Additionally, quotations from the transcribed text were provided to illustrate the relationship between the data and the findings. The research findings, including background information on mothers and infants, are reported as accurately as possible to allow readers to determine the transferability of the findings to a specific context. Also, data saturation enhanced the study's trustworthiness [33].

## 4 Findings

Interviews were conducted with 25 mothers whose infants (*n* = 26) were being treated in the NICU. One mother who signed informed consent declined to be interviewed because the infant recovered quickly and the mother felt that she did not have enough insight into the pain relief interventions to participate in the interview. Background information on mothers and newborns is shown in Tables 2 and 3.

**TABLE 2:** Background information on mothers participated in the study (*n* = 25).

Characteristics	*n*	%
Age in years (mean 32.0, SD = 6.3, range 21–46)		
< 30	9	36
30–35	8	32
36–40	6	24
> 40	2	8
Education		
No vocational education	5	20
Vocational education	9	36
Polytechnic education^a^	7	28
University education	4	16
First time mother		
Yes	9	36
No	16	64
Distance from home to hospital (km) (mean 43.7, SD = 49.6, range 2–160)		
< 10	8	32
10–50	10	40
> 50	7	28
Opportunity to participate in infant's care in the NICU		
Every day	23	92
Almost every day	2	8
Experience with glucose for pain relief before the RCT^b^		
No previous experience	4	16
Was used for the previous child	8	32
Was used to this infant	12	48
Experience from other context	1	4

^a^ Focuses on practical education, typically provides bachelor's degrees.

^b^ All infants were participants in a randomised controlled trial of the effectiveness of non‐pharmacological pain relief methods during a heel lance procedure.

**TABLE 3:** Background information on infants (*n* = 26) whose mothers participated in the study.

Characteristic	*n*	%
Gestational age at birth in weeks (mean 36 + 4, SD 3.0, range 32 + 2–40 + 5)		
< 34	6	23
34–36 + 6	9	35
≥ 37	11	42
Postnatal age at time of last study^a^ in days (mean 8.8, SD = 2.8, range 2–16)		
< 5	14	54
5–10	8	31
> 10	4	15
Birthweight in grams (mean 2995.4, SD = 904.1, range 1090–4240)		
< 250 0	7	27
2500–3500	9	35
> 3500	10	38
Sex		
Female	9	35
Male	17	65
Primary reason for admission to NICU		
Prematurity	10	38
Respiratory distress	14	54
Other neonatal disease	2	8
Heel lances before the RCT (mean 12.7, SD = 6.3, range 2–26)		
< 10	8	30
10–15	9	35
> 15	9	35

^a^ All infants were participants in a randomised controlled trial of the effectiveness of non‐pharmacological pain relief methods during a heel lance procedure.

### 4.1 Use of Oral Glucose for Pain Relief

The mothers' views on the use of oral glucose as a method of pain relief in critically ill infants consisted of four main categories: (1) varying perceptions of acceptability, (2) an easy‐to‐implement method, (3) contradictory opinions on effectiveness and (4) another method would be better (Table 4).

**TABLE 4:** Mothers' experiences with oral glucose for pain relief in critically ill infants.

Main category	Generic category	Sub‐category
Varying perceptions of acceptability	Strange method for pain relief	Glucose is known to be harmful to infants
		Scepticism about the effectiveness of glucose
	Acceptable to use for pain relief	Glucose is acceptable if it relieves infant's pain
		Glucose is a mild and harmless pain relief method
		Glucose is an option when parents cannot participate in pain relief
An easy‐to‐implement method	Easy to administer	Glucose is readily available
		Glucose is easy to dose into infant's mouth
	Anyone can carry out	Glucose could be given by professionals
		Glucose could be administered by parents
Contradictory opinions on effectiveness	Beneficial for the infant	The infant was willing to take glucose solution
		Glucose diverted the infant's attention away from the pain
		Glucose relieved infant's pain
	Did not work as expected	Glucose was not to the infant's liking
		The infant aspirated glucose
		Glucose relieved pain for a short time
Another method would be better	Mother‐led pain relief more recommended	Glucose lacks maternal closeness
		Mother's breast milk could be a better method
	Insufficient alone	Glucose should be combined with non‐nutritive sucking
		Glucose should be combined with comforting care

#### 4.1.1 Varying Perceptions of Acceptability

This main category was composed of two generic categories: (1) strange method for pain relief, and (2) acceptable to use for pain relief.

Mothers found it strange that oral glucose was used to treat pain in newborns because sugar is known to be harmful to young children. They were concerned about the idea of giving oral glucose to newborns because they considered it common knowledge that sugar is not good for anyone's health. Many mothers also considered the connection between dental health and the use of oral glucose for pain relief. Some mothers thought the use of glucose was outdated.

I've been a bit wary of sugar ever since I saw it being given to babies. But on the other hand, I've already gotten used to it. But I am somewhat against the use of sugar, when we try to keep it to a minimum for babies and small children. And then sugar is given. Even though what they need is a very small amount, still I guess that's my impression. M016

The mothers were surprised to learn that administering oral glucose can relieve procedural pain in infants. They had not considered giving glucose as a treatment option. Some were sceptical about its pain‐relieving effect but later found that it was effective.

It sounded a bit funny. I thought it was a joke at first, when they said it is candy day. But then, when I saw how it works, it was clear that the baby always calmed down. M026

Mothers thought it was acceptable to use oral glucose for pain relief during procedures and examinations if it alleviated the newborn's pain or otherwise helped the infant. They considered using oral glucose acceptable, especially in cases where parental involvement in pain management was not possible. Some mothers also noted that they would only use glucose in the hospital, not at home, to treat their infant's pain.

So of course I think it would be really good if a parent can be present. But I don't really know if sugar has been found to be effective, so what about that? Of course, in, for example, situations where parents are able to be there why not have both [sugar and parents]? But, but just the fact that if there is nothing else available, sugar is really good. M023

Based on the mothers' perceptions, glucose was a mild and harmless method of pain relief, and they preferred it to painkillers. Some also felt that sugar was gentle for newborns because of its sweet taste, which is reminiscent of mother's milk.

I'd rather give [the baby] sugar than some painkillers. I feel like it's a lighter version of that [painkillers], it's like getting something more like milk or something delicious like that. M004

#### 4.1.2 An Easy‐To‐Implement Method

This main category consisted of two generic categories: (1) easy to administer, and (2) anyone can carry out.

According to the mothers, oral glucose was easy to administer because it was readily available. They had noticed a syringe of glucose on a table near their newborn's bed and had seen it used in different situations. Oral glucose was also given to their infants in the maternity ward during examinations. The mothers felt that administering oral glucose was fast and easy by putting it in the infant's mouth with a syringe or pacifier.

I would guess that it [oral glucose] would always be available. All you have to do is take it from the lab trolley and then put it on the baby's pacifier or, if there is no pacifier, in their mouth. It doesn't take long. M001

The mothers believed that anyone involved in the care of the newborn could carry out pain relief with oral glucose. Typically, the infant's nurse or doctor administered the glucose. Laboratory nurses did not administer the glucose solution, but the mothers believed that laboratory nurses could do so if the infant's nurse was unavailable. The mothers felt that parents could also administer oral glucose in addition to professionals. Some of the mothers had administered glucose to their infants during the infant's care. However, the mothers felt that they needed guidance to administer the glucose.

Certainly, parents can give it [oral glucose], if they are taught how much and how to do it. M026

#### 4.1.3 Contradictory Opinions on Effectiveness

This main category consisted of two generic categories: (1) beneficial for the infant, and (2) did not work as expected.

Many infants benefited from receiving glucose during painful procedures. Their mothers felt that the infants were pleased to take the glucose solution and seemed to like its taste. Some infants sucked on the solution for a long time and looked satisfied. The mothers thought that the glucose diverted their infant's attention away from the pain.

I have noticed that when you give him sugar, he likes it. That is perhaps the kind of thing that takes his mind elsewhere. And at the same time, it's a nice thing to focus on instead of thinking about the pain. M025

Most mothers found that glucose relieved infant pain during blood sampling. They noticed that their infants showed no signs of pain during a heel lance, remained calm throughout the procedure and stopped crying when the glucose solution was put in their mouths. Some mothers had experience with having their newborns' blood sampled with and without glucose and felt that the analgesic effect of glucose was clear.

The baby's pain [during the blood sampling] was really intense on the other side [in the maternity ward] compared to this, when there was no pain relief, not even a pacifier in the maternity ward. M020

Some mothers felt that glucose did not work as expected in infants' pain management. They reported that their infants did not like the taste of the glucose and by grimacing and pushing it out of their mouth. Some mothers speculated that their newborns' state of arousal might have caused them to force the glucose solution out of their mouths instead of swallowing it. One infant aspirated the solution and started coughing. The mother was surprised by her infant's reaction because on previous occasions, the glucose solution had not caused any harm. For some infants, the mothers found that glucose was only effective in the short term. One mother wondered if frequent glucose administration had habituated her infant, so the glucose no longer relieved his pain.

But it [oral glucose solution] may not be that long acting. As in it works for a while and then the baby might be restless again. M027

#### 4.1.4 Another Method Would Be Better

This main category included two generic categories: (1) mother‐led pain relief more recommended and (2) insufficient alone.

According to the mothers' views, glucose was not considered the best method of pain relief. They preferred mother‐led methods, in which maternal involvement is an essential part of pain relief, as they believed their own involvement would benefit their newborns more. Many mothers found that glucose alone was like a medicine, providing no maternal closeness to the infant. They felt that a mother's presence and touch would provide infants with comfort and security and enable mother–infant interaction.

Personally, I feel that this kind of personal involvement from a mother is a better alternative to sugar, if you can somehow bring a sense of security to the baby in that moment of pain. M011

Mothers suggested breast milk as a substitute for glucose. Many wondered if their breast milk would be a better method of pain relief than a glucose solution. They had already pumped milk for their infants, so it would have been available in the same way as glucose. The mothers felt that breast milk would be a more natural analgesic than glucose and would therefore prefer it if it was equally effective. Some mothers were also familiar with the analgesic effects of breast milk. They had given milk to infants who spat out glucose or otherwise indicated that they did not like it.

I didn't notice any difference in effectiveness [between glucose and milk drops] when I gave it to the baby myself, but I'm always in favor of anything natural. So maybe I'd prefer just breastmilk if there is not a big difference between the two. M022

According to several mothers, oral glucose alone was not sufficient for pain relief, and they recommended combining it with other methods. Some suggested combining glucose with non‐nutritive sucking. They had seen this combination used in their infants' care and thought the newborns happily sucked on the sweetened pacifier. Some mothers thought infants needed human closeness. They believed glucose could be combined with the presence and touch of the nurse caring for the infant. Additionally, mothers suggested combining verbal comfort with oral glucose.

I hope that there is a nurse present during the blood sampling and the baby is not alone, even if sugar is given. I feel that by touching or talking or something like that, you can communicate that you are there. Otherwise, the baby might feel alone and just getting poked. M004

## 5 Discussion

This study provided new insights into mothers' perceptions on the use of oral glucose to alleviate neonatal pain in the NICU. Mothers' involvement in pain relief, such as being present, implementing pain relief methods and advocating for them, is an essential part of neonatal pain management [8, 12]. Previous studies on non‐pharmacological methods of neonatal pain relief have primarily examined their effectiveness [18, 34] and the role of parental involvement in pain management [35]. Although sweet solutions are a widely used method for relieving newborn pain, previous studies have shown that mothers rarely use them [22, 23].

Our study found that many mothers considered the use of oral glucose for neonatal pain relief strange. Some were sceptical about the analgesic effect of glucose; however, most of the confusion was related to awareness of dietary recommendations for infants, which include avoiding sugar. These results align with those of a Canadian study [8] that found parents were sceptical about using a sweet‐tasting solution, oral sucrose, to relieve pain in healthy infants. The parents mentioned that sucrose is an unnecessary sugar that could harm the digestive system of newborns [8]. In our study, mothers accepted the use of oral glucose for neonatal pain management if it alleviated their infant's pain. They viewed it as a less harmful alternative to painkillers and a good option when they were unable to participate in their infants' pain management. Hughes et al. found that informing parents about the analgesic effects and side effects of sucrose alleviated their concerns about using it. Some parents chose sucrose as a pain relief method for their infants instead of SSC or breastfeeding. They justified their choice based on the strong evidence‐based effectiveness of sucrose [8]. Based on our findings and previous research, it might be useful to discuss mothers' perceptions and provide information on the benefits and side effects of sweet solutions when guiding mothers on the use of sweet solutions for pain management.

Our study showed that the mothers had positive perceptions implementing glucose solution as a pain relief method. They felt that it was readily available and accessible in all situations. Some mothers had given glucose to their infants during procedures or treatments. Even mothers who had not yet administered glucose expressed that it is also suitable for pain management by the parents with proper guidance. However, based on previous studies, pain management using sweet solutions administered by parents does not appear to be common in NICUs [23, 24, 25]. Currently, sweet solutions are often classified as pharmacological agents [36], and their use may be subject to hospital‐specific guidelines [25]. However, some evidence suggests that, with adequate guidance, pharmacological treatment by parents is effective and safe [37].

Our study found that mothers had mixed personal perceptions of the pain‐relieving effects of glucose. While many were satisfied with its effectiveness, others felt that glucose either did not relieve pain or did so only briefly. These views align with previous research indicating that the analgesic effect of sweet‐tasting solutions lasts approximately 4 min [38], and for some infants, the sweet‐tasting solution was not effective in minimising pain [39]. On the other hand, the perception of the poor effectiveness of glucose may have been related to the phenomenon described by mothers whose infants forced glucose out of their mouths. However, there is evidence that a very small amount, 0.1 mL of sucrose, is an effective dose [39].

According to our research, mothers do not consider glucose to be the most recommended method for relieving neonatal pain. They thought that infants need comforting by their mother during painful procedures, and that glucose is just a painkiller lacking human touch and interaction. Several mothers suggested using breast milk instead of glucose. They believed that breast milk would naturally and sweetly relieve the newborn's pain and involve the mother in pain management. Previous research has demonstrated that mothers have a strong desire to help their infants [6]. The use of glucose alone for pain relief may not offer mothers the same sense of involvement as methods that promote maternal participation or facilitate direct mother–infant interaction. A recent systematic review indicates that pumping breast milk for a hospitalised infant is meaningful to mothers because it alleviates their sense of guilt, creates an emotional connection with their infant and symbolises their maternal role [40]. However, there is not yet enough research evidence on the effectiveness of breast milk for neonatal pain management [41], which may explain why it is rarely used instead of glucose or sucrose.

### 5.1 Limitations

Despite the significant findings, the study has some limitations. First, selection bias may have occurred because mothers who considered parental involvement in their infant's pain relief important may have been more likely to participate in the study. Additionally, only mothers of NICU infants in Finland participated in the interviews, so it is unclear whether the findings are applicable to other countries or settings. Nevertheless, we have presented the backgrounds of mothers and their newborns in as much detail as possible to enable readers to evaluate the transferability of the results.

### 5.2 Recommendations for Practice

Based on mothers' views, oral glucose is an easy‐to‐use method of pain relief in the NICU. It can be administered by healthcare professionals as well as parents. It is particularly suitable for use in situations where the parents are not present to provide comfort, or where rapid pain relief is needed. However, mothers also identified some weaknesses in using glucose for pain relief; it lacks the closeness of a mother or caregiver, some newborns do not like the taste of glucose and its analgesic effect is not always sufficient. Mothers suggested that orally administered breast milk could be a suitable alternative to glucose, as it is both sweet and perceived as more natural. Our results highlight the importance of recognising that mothers' knowledge and experience with glucose is unique and influences their preference for using it to relieve their infants' pain. In line with the principles of family‐centred care, mothers should receive evidence‐based information about oral glucose, alternative pain relief options and ways they can participate in their infant's pain management. They should also be involved in the decision‐making regarding the pain management of their critically ill infants.

## 6 Conclusion

This study provides valuable information about mothers' views using oral glucose for procedural pain management in their critically ill newborns. It provides a novel perspective on the use of sweet solutions in neonatal pain management, which differs from previous studies that have mainly focused on the efficacy and prevalence of sweet solutions. Mothers felt that the strength of glucose as a pain relief method was its ease of use. However, it was not as effective as expected for all infants, and it lacked maternal closeness. They recommended combining glucose with another method or adopting a mother‐led approach to improve pain management. The study's findings can be used to improve outcomes for families and newborns in the NICU.

## Author Contributions

Critical contributions to the study design: Anna‐Kaija Palomaa and Tarja Pölkki. Data collection: Anna‐Kaija Palomaa. Preliminary analysis: Anna‐Kaija Palomaa. Final analysis: Anna‐Kaija Palomaa, Tarja Pölkki, Eeva Talus and Sirpa Keskitalo‐Leskinen. Writing the manuscript: Anna‐Kaija Palomaa. Reviewing and editing the manuscript: Anna‐Kaija Palomaa, Tarja Pölkki, Eeva Talus and Sirpa Keskitalo‐Leskinen. All authors meet the authorship criteria and agree with the content of the manuscript.

## Funding

This work was supported by the Stiftelsen Alma och K. A. Snellman Säätiö and Päivikki ja Sakari Sohlbergin Säätiö.

## Ethics Statement

The study (ref: 296/2018) received ethics approval on 20‐03‐2023, from the regional medical research ethics committee of the North Ostrobothnia Wellbeing Services County. The babies of the mothers participated in an RCT study. The registration number for this study is ClinicalTrials.gov NCT04967118.

## Consent

The participants signed an informed consent document, which included consent for audio‐recorded interviews.

## Conflicts of Interest

The authors declare no conflicts of interest.

## Supporting information


**Table S1:** Background information on the authors at the time of the study.

**Table S2:** COREQ checklist.

## Data Availability

The data that support the findings of this study are available on request from the corresponding author. The data are not publicly available due to privacy or ethical restrictions.
