# Supplementary material for: Mothers' Views on the Use of Oral Glucose for Pain Relief in a Neonatal Intensive Care Unit
Source: Nurs Crit Care. 2026 Jan 8;31(1):e70318. doi: 10.1111/nicc.70318 (PMC12781963; doi:10.1111/nicc.70318)
Supplement: Supplementary file 1 — Table S1: Background information on the authors at the time of the study. [file NICC-31-0-s002.docx]

Table S1 Background information on the authors at the time of the study

| Author | Background |
| --- | --- |
| The first author | Female  Neonatal nurse, RN  She works as an assistant head nurse in the NICU  She was on work leave during the study  She has conducted qualitative research during her master's studies and her current doctoral studies |
| The second author | Female  Neonatal nurse, RN  She works as a Clinical Nurse Specialist and was employed part-time in the NICU at the time of study  She has conducted qualitative research during her current doctoral studies. |
| The third author | Female  Neonatal nurse, RN  She was working as a Nursing instructor in the NICU at the time of the study  She was trained in qualitative research as part of her master’s studies |
| The fourth author | Female  Register Nurse, PhD  She works as Professor of Nursing Science at the University of Oulu  She is specialized in research on pain management, and child, adolescent, and family-centered care  She has strong methodological skills in qualitative research |
